# Novel Enzyme Shows Potential as an Anti-HIV Target

**DOI:** 10.1371/journal.pbio.0030074

**Published:** 2005-02-08

At just 9.8 kilobases, the HIV genome pales in comparison to the 3.2 gigabases of its human and nonhuman primate targets. The compact retrovirus encodes just 14 proteins, which play different roles in promoting viral infection and virulence. As a retrovirus, HIV uses the host's cellular machinery—including RNA polymerases, which carry out transcription—to copy its RNA genome into DNA and infiltrate human chromosomal DNA. Once the virus is integrated (now called a provirus), its genes can be transcribed.

Adept as HIV is in exploiting its host's molecular resources, the virus can't establish a foothold without the services of its skeleton crew. The HIV transcription factor Tat (“transactivator of transcription”), for example, is an essential regulator of HIV gene expression. Without Tat, HIV transcripts don't reach full length and can't effect viral replication. In a new study, Melanie Ott and colleagues identify an enzyme that regulates viral transcription by modifying Tat.

The regulation of HIV genes depends on a complex interplay between proviral DNA, cellular proteins and transcription factors, and Tat. Unlike most transcription factors, Tat activates transcription by binding to RNA, specifically to a bulging “stem-loop” structure that forms at one end of all viral transcripts called the *trans*-acting responsive element (TAR). Tat binding to TAR requires recruiting the enzyme cyclin-dependent kinase 9 (CDK-9) to the HIV promoter (where transcription begins). CDK-9 chemically modifies the RNA polymerase and enhances its transcribing efficiency.

The transcription process—including the labyrinthine protein–protein and protein–DNA (and in the case of Tat, protein–RNA) interactions—is highly regulated. One process that figures prominently in this regulation is acetylation, which adds an acetyl group (a molecule made of oxygen, hydrogen, and carbon) to a molecule or protein. Histone acetylation was long thought to influence transcription by regulating the structure and function of chromatin, which is an assembly of proteins (mostly histones) and DNA. Another, more widely accepted model proposes that histone acetylation controls transcription by recruiting cofactors required for transcription.

Acetylation and deacetylation enzymes can also target other proteins. Of the three classes of deacetylases known to modify human histones, the sirtuins (SIRT1–7) appear to preferentially target a number of nonhistone proteins. Ott and colleagues first tested the ability of all seven SIRT proteins to deacetylate Tat by placing them in a test tube with Tat proteins. Though three SIRT enzymes caused Tat acetylation, only one, SIRT1, is a nuclear enzyme, like Tat, suggesting that SIRT1 might work similarly in living cells.[Fig pbio-0030074-g001]

**Figure pbio-0030074-g001:**
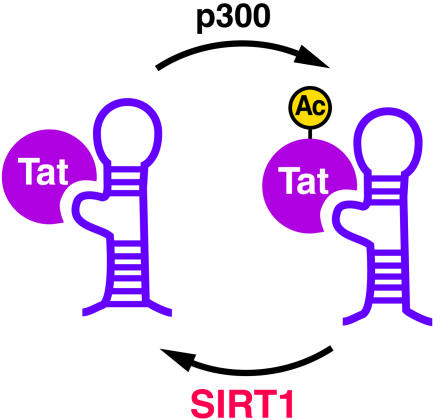
Recycling of Tat through deacetylation by SIRT1

Ott and colleagues went on to show that transcription via Tat occurs in the presence of SIRT1, but not when SIRT1's catalytic center is removed. Experiments using cells taken from transgenic mice lacking SIRT1 demonstrated that introducing human SIRT1 enzymes increased Tat's transcriptional effects in a dose-dependent manner, while treating cells with the small molecule HR73, a derivative of a molecule that inhibits the yeast version of the SIRT1 protein, caused a 5-fold reduction in HIV transcription.

The authors propose a cycle of transcriptional transactivation in which SIRT1 deacetylates Tat at the HIV promoter. Deacetylated Tat associates with CyclinT1 and TAR, and leads to transcription. Tat acetylation dissociates Tat from CyclinT1 and TAR, and transfers Tat to the elongating polymerase complex. Since acetylated Tat can't recruit CyclinT1 and CDK-9, the authors explain, a new round of transcription requires that new, unacetylated Tats are produced or existing Tats are deacetylated. Thus, efficient viral replication depends on adequate Tat supplies. And since HIV gene expression relies on SIRT1's enzymatic activity, inhibiting SIRT1 could prove to be a promising anti-HIV therapy. Future study will have to verify whether inhibiting SIRT1 can successfully put the brakes on HIV transcription and control the virus (See also “A New Paradigm in Eukaryotic Biology: HIV Tat and the Control of Transcriptional Elongation” [DOI: 10.1371/journal.pbio.0030076]).

